# Transcription Factors Indirectly Regulate Genes through Nuclear Colocalization

**DOI:** 10.3390/cells8070754

**Published:** 2019-07-20

**Authors:** Zhiming Dai

**Affiliations:** 1 School of Data and Computer Science, Sun Yat-Sen University, Guangzhou 510006, China; daizhim@mail.sysu.edu.cn; 2 Guangdong Province Key Laboratory of Big Data Analysis and Processing, Sun Yat-Sen University, Guangzhou 510006, China

**Keywords:** nuclear architecture, gene regulation, transcription factor

## Abstract

Various types of data, including genomic sequences, transcription factor (TF) knockout data, TF-DNA interaction and expression profiles, have been used to decipher TF regulatory mechanisms. However, most of the genes affected by knockout of a particular TF are not bound by that factor. Here, I showed that this interesting result can be partially explained by considering the nuclear positioning of TF knockout affected genes and TF bound genes. I found that a statistically significant number of TF knockout affected genes show nuclear colocalization with genes bound by the corresponding TF. Although these TF knockout affected genes are not directly bound by the corresponding TF; the TF tend to be in the same cellular component with the TFs that directly bind these genes. TF knockout affected genes show co-expression and tend to be involved in the same biological process with the spatially adjacent genes that are bound by the corresponding TF. These results demonstrate that TFs can regulate genes through nuclear colocalization without direct DNA binding, complementing the conventional view that TFs directly bind DNA to regulate genes. My findings will have implications in understanding TF regulatory mechanisms.

## 1. Introduction

Transcription factor (TF) binds DNA recognition sites in genomic regulatory regions to control genomic transcription. TF is critical in transcriptional regulation. Generally, there are two main methods to determine genome-wide TF direct targets. Chromatin immunoprecipitation followed by microarray hybridization (ChIP-chip) is a wonderful tool for studying direct physical information of binding between TF and DNA regions [1,2,3]. In higher organisms, particularly mammals, chromatin immunoprecipitation coupled with massively parallel sequencing (ChIP-seq) is a widely used method for mapping direct physical TF-DNA interactions genome-wide [4,5]. ChIP-chip and ChIP-seq assays are effective in uncovering genome-wide maps of the locations bound by the immunoprecipitated TF. However, profiling multiple TFs in a cell type using either of two assays is costly and requires large input cell numbers. An alternative to experimental high-throughput profiling of TF-DNA interactions is digital TF footprinting, in which TF-DNA interactions are computationally inferred by integrating chromatin accessibility patterns with the underlying TF bound motifs. The chromatin accessibility patterns can be determined by DNase-seq (Digestion with the nuclease DNase I, coupled to high-throughput sequencing), ATAC-seq (Transposase-Accessible Chromatin followed by sequencing) and FAIRE-seq (Formaldehyde-Assisted Isolation of Regulatory Elements followed by sequencing) [6,7,8,9,10,11,12]. These technologies have been applied to determine the Encyclopedia of DNA Elements (ENCODE) in various human cell lines [13].

Another way to reveal regulatory relations between TF and its target genes is to measure gene expression changes in response to TF perturbation [14,15]. However, TF perturbation experiments cannot distinguish direct relations from indirect ones. TF perturbation experiments and ChIP-chip (or ChIP-seq) experiments serve as complementary data sources to uncover TF cellular functions. Hu et al. have knocked out 269 budding yeast TFs one at a time, and measured genome-wide gene expression changes [16]. The differentially expressed gene targets were compared with the TFs direct bound gene targets measured by ChIP-chip [17]. Only ~3% of TF knockout affected genes (referred to as TF-KO genes) are bound by the corresponding TF. A possible explanation for the small overlap is that TFs can regulate indirect bound targets via regulatory cascades. However, removal of indirect TF-target regulatory interactions resulted in negligible improvements to the overlap [16]. Reimand et al. have reanalyzed the TFs knockout expression data and identified more TF-KO genes that are bound by the corresponding TF—four times the total reported by Hu et al. [18]. However, the overlap between TF-KO genes and TF bound targets is still very small.

Over the past few years, high-throughput chromatin conformation capture assays have generated three-dimensional architecture of whole genomes for eukaryotic organisms [19,20,21,22,23,24,25,26,27]. Three-dimensional genome structure is crucial for gene regulation and cell functions. Genome regions with similar chromatin states (e.g., histone modifications) cluster within the nucleus [28,29]. Genes showing differentially expressed upon knockout of the same TF tend to show nuclear colocalization [30]. Spatially proximal genes tend to show co-expression [31,32]. Functional gene groups are concentrated in the nuclear space of the human genome [33]. 

In mammals, TFs can regulate gene transcription by binding distal regulatory regions (e.g., enhancers) [34,35,36]. These distal regulatory regions can be brought into proximity to target genes by intra-chromosomal interaction [37,38,39]. It is interesting to test whether this regulatory mode exists in lower eukaryotes. I hypothesized that a possible explanation for the small overlap between TF-KO genes and TF direct bound gene targets is that knockout of the TF affects genes that are spatially adjacent to genes directly bound by the corresponding TF. Analyzing genome-wise TF binding data, TF knockout data and three-dimensional genome structure data in budding yeast Saccharomyces cerevisiae, I found that a statistically significant number of TF-KO genes show nuclear colocalization with genes directly bound by the corresponding TF. TFs can regulate their unbound genes by being in the same cellular component with TFs that directly bind these genes. 

## 2. Materials and Methods

### 2.1. Data Preparation 

Genome-wide binding affinity data corresponding to 203 TFs were taken from Harbison et al. [17]. A P value cutoff of 0.005 was used to define the set of genes bound by a particular TF. Combining several motif finding methods, Harbison et al. also identified DNA motifs that are not bound by the corresponding TFs in each promoter [17]. Genome-wide changes in gene expression data corresponding to the knockout of 269 TFs were taken from Hu et al. [16]. Differentially expressed genes for each TF knockout were identified by Hu et al. [16] and Reimand et al. [18], respectively. As the identification method of Reimand et al. were reported to have improvement compared with that of Hu et al., I used the list of differentially expressed genes from Reimand et al. The TF knockout data and TF binding data have 188 TFs in common. For TF-KO genes of each TF, I excluded those genes that are either bound genes of the corresponding TF or close to (< 3k bp) bound genes of the corresponding TF. For TF bound genes of each TF, I also excluded those genes that are either TF-KO genes of the corresponding TF or close to (< 3k bp) TF-KO genes of the corresponding TF. In this way, for each TF, there are no genes that are overlapped and linear proximity between its knockout affected genes and its bound genes. After these exclusions, I restricted the analysis to TFs with more than 20 knockout affected genes and bound genes, respectively, resulting in a total of 136 TFs. In this study, I focused on these 136 TFs. 

Genome-wide inter-chromosomal and intra-chromosomal interaction data were taken from Duan et al. [19]. As in the original literature, a false discovery rate (FDR) value cutoff of 0.0001 was used to define inter-chromosomal and intra-chromosomal interactions. There were 66,127 inter-chromosomal interactions and 37,089 intra-chromosomal interactions among 3991 segments on different chromosomes, with kilo-base resolution, which have been identified. ~20% of all intra-chromosomal interactions are those between segments separated by < 60k bp. In the original literature, intra-chromosomal interactions that are between segments separated by < 20k bp were eliminated. In this study, I set a stricter cutoff to control for the interactions caused by linear proximity in the genome. I eliminated intra-chromosomal interactions in which the two segments are separated by < 60k bp. Two segments that interact with each other are colocalized in the nucleus. For each gene, I identified the segments that are close to it (within 2.5k bp to the transcription start site (TSS)). This cutoff value was chosen according to the original literature. Most genes have their corresponding segments. In this way, I could evaluate whether gene pairs are colocalized in the nucleus according to colocalized segment pairs. I referred to the TF-KO genes, which show nuclear colocalization with genes bound by the corresponding TF, as TF spatially adjacent regulated genes (referred to as TF SAR genes), and referred to the corresponding TFs as SAR TFs. I referred to the TF-KO genes, which do not show nuclear colocalization with genes bound by the corresponding TFs, as TF non-SAR genes. The TSS data was taken from David et al [40].

Cellular component and biological process data were taken from GO database [41]. The number of genes in the same cellular component ranges from 1 to 2341. The number of genes in the same biological process ranges from 1 to 1103. For all pairs between TFs and their SAR genes, I calculated the frequency of pairs in which the SAR TFs are in the same cellular component with TFs that directly bind the TF SAR genes. For all pairs between TFs and their SAR genes, I calculated the frequency of pairs in which the TF SAR genes are involved in the same biological process with any genes bound by the corresponding SAR TFs. For all pairs between TFs and their spatially non-adjacent regulated genes, I calculated the frequency of pairs in which the TF non-SAR genes are involved in the same biological process with any genes bound by the corresponding SAR TFs.

Genome-wide gene expression data used for co-expression analysis were measured under normal growth conditions [42,43,44], a total of 112 time points. I calculated Pearson expression correlation coefficient between TF SAR genes and genes bound by the corresponding TFs. I also calculated Pearson expression correlation coefficient between TF spatially non-SAR and genes bound by the corresponding TFs. For each TF, I calculated pair-wise Pearson expression correlation coefficient among its bound genes that show nuclear colocalization with its knockout affected genes. For each TF, I also calculated pair-wise Pearson expression correlation coefficient among its bound genes that do not show nuclear colocalization with its knockout affected genes. Pearson expression correlation coefficient was calculated by using “corr” function in Matlab.

Genome-wide nucleosome occupancy data were taken from Lee et al. [45]. We compared nucleosome occupancy profiles in promoter regions between TF bound genes and TF SAR genes. To avoid confusion, we excluded genes that are both TF bound genes and TF SAR genes in the analysis of nucleosome occupancy data.

### 2.2. Randomized Experiments

I used randomized experiments to test the statistical significance of frequency of TF-KO genes showing inter- and intra-chromosomal colocalization with genes bound by the corresponding TFs. For each TF, I generated a random gene for each of its knockout affected genes. Considering that TF regulation shows preference for some specific chromosomes, the random gene should be on the same chromosome as the actual gene. In this way, for each TF, I generated a set of random genes, the number of which was the same as the actual number of its knockout affected genes. Similarly, for each TF, I generated a set of random genes, the number of which was the same as the actual number of its bound genes. For each pair between one TF and its random knockout affected gene, I determined whether the gene shows nuclear colocalization with any random genes bound by the corresponding TF. For all pairs, I calculated the frequency of pairs that show this property of nuclear colocalization. If the nuclear colocalization with TF bound genes is not the feature of actual TF-KO genes, the random pairs should show a similar degree of frequency as actual pairs. I repeated the randomized experiment 10,000 times to calculate the frequency of experiments having higher colocalized frequencies than that of actual pairs between TFs and their knockout affected genes, and referred to this value as the P value. I also used similar randomized experiments to test the statistical significance of frequency of TF bound genes showing inter- and intra-chromosomal colocalization with genes whose gene expression are significantly affected by knockout of the corresponding TFs.

I used randomized experiments to test the statistical significance of frequency of TF SAR genes showing cellular co-component between their SAR TFs and their binding TFs. For each TF, I generated a random gene for each of its SAR gene. In this way, for each TF, I generated a set of random genes, the number of which was the same as the actual number of its SAR gene. For each pair between one TF and its random SAR gene, I determined whether the TF is in the same cellular component with any TFs that directly bind the corresponding gene. For all pairs, I calculated the frequency of pairs that show this property of co-component. I repeated the randomized experiment 10,000 times to calculate the frequency of experiments having higher co-component frequencies than that of actual pair between TFs and their SAR genes, and referred to this value as *P* value.

In this study, all computational methods and analyses were implemented using Matlab. The source codes are on https://github.com/daizhim/Transcription-factors-indirectly-regulate-genes-through-nuclear-colocalization/tree/master/upload.

### 2.3. Statistical Method

Given two samples of values, the Mann-Whitney U-test is designed to examine whether they have equal medians. The main advantage of this test is that it makes no assumption that the samples are from normal distributions. Mann-Whitney U-test was implemented by using “ranksum” function in Matlab. 

## 3. Results

### 3.1. TF-KO Genes Show Nuclear Colocalization with Genes Bound by the Corresponding TF

To investigate the spatial relationship between TF-KO genes and TF direct bound genes, I used three genome-wide yeast data sets. One is a compendium of 269 TF knockout genome-wide microarrays [16]. A previous study has identified differentially expressed genes for each TF knockout [18] with improvement compared with the original study [16]. I used these genes and referred to them as knockout affected genes for each TF (i.e., TF-KO genes). Another data set is genome-wide DNA binding data of 203 TFs [17]. I focused on TFs whose knockout data and binding data are both available, resulting in a total of 188 TFs. To avoid confusion, for TF-KO genes, I excluded those genes that are either bound genes of the corresponding TF or close to (<3k bp) bound genes of the corresponding TF. For TF bound genes, I also excluded those genes that are either knockout affected genes of the corresponding TF or close to (<3k bp) knockout affected genes of the corresponding TF. In this study, I restricted the analysis to TFs with more than 20 knockout affected genes and bound genes, respectively. The third data set is the genome-wide inter-chromosomal and intra-chromosomal interactions data [19]. Chromosomal interaction partners were determined with kilo-base resolution for nearly 4000 segments on different chromosomes.

**Figure 1:**
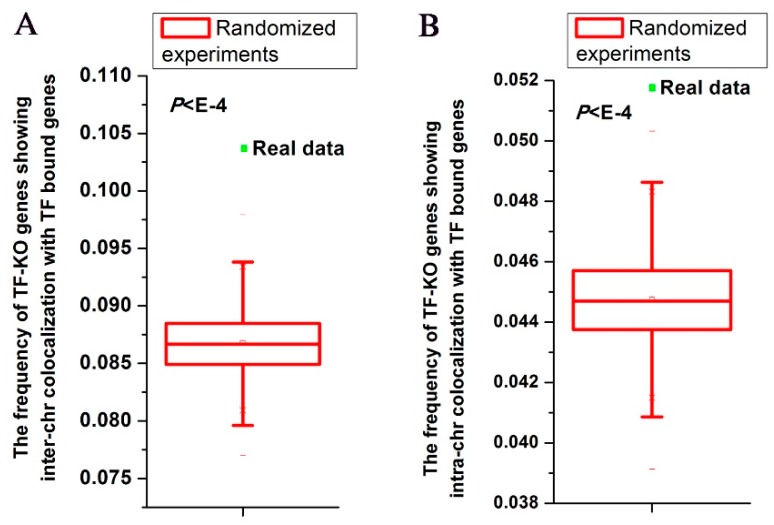
For all transcription factors (TFs), a statistically significant number of TF-KO genes show nuclear colocalization with genes bound by the corresponding TF. Distributions of the frequencies of pairs between TF and its knockout affected gene that show inter-chromosomal (i.e., inter-chr) colocalization (**A**) and intra-chromosomal (i.e., intra-chr) colocalization (**B**) with genes bound by the corresponding TF. The dots were for the realistic data (3967/38,359 = 10.34%, 1987/38,359 = 5.18%), while the box plots depicted the distributions for 10,000 randomized experiments. The statistical significance was indicated.

First, I examined whether TF-KO genes show nuclear colocalization with TF direct bound genes using inter-chromosomal interactions data. I generated random gene pairs to test the statistical significance (see details in Materials and Methods section). I repeated the randomized experiment 10,000 times to calculate P value. For all TFs, the actual frequency (~10%) of TF-KO genes showing inter-chromosomal interactions with genes bound by the corresponding TF was higher than those of all randomized experiments (*P* < 10^−4^, Figure 1A). The inter-chromosomal interaction frequencies of individual TFs ranged from ~0% to ~28% (Table S1). 

Second, I tested spatial relationship between TF-KO genes and TF direct bound genes using intra-chromosomal interactions data. To control for linear proximity in the genome, I only considered intra-chromosomal interactions that are separated by at least 60k bp. For all TFs, ~5% of TF-KO genes showed intra-chromosomal interactions with genes bound by the corresponding TF, which was higher than those of all randomized experiments (*P* < 10^−4^, Figure 1B). The intra-chromosomal interaction frequencies of individual TFs ranged from 0% to ~17% (Table S1).

Third, I examined whether my results are robust to the choice of data. I used another list of TF-KO genes identified by another computational method [16]. TF-KO genes still had a statistically significant tendency to show nuclear colocalization with genes bound by the corresponding TF, though the significance of intra-chromosomal interactions became weaker (*P* < 10^−4^ for inter-chromosomal interactions, *P* = 0.0021 for intra-chromosomal interactions, Figure S1).

Fourth, I asked whether TF direct bound genes show nuclear colocalization with genes affected by knockout of the corresponding TF. However, I found that the enrichment of nuclear colocalization was weak compared with those of randomized experiments (*P* = 0.72 for inter-chromosomal interactions, *P* = 0.64 for intra-chromosomal interactions, Figure S2).

Taken together, I showed that a considerable number of TF-KO genes show nuclear colocalization with genes bound by the corresponding TF. This result indicated that TFs are spatially adjacent to a fraction of their knockout affected genes, though the TFs do not directly bind these genes. In the following analysis, I referred to TF-KO genes that are spatially adjacent to the corresponding TF bound genes as TF spatially adjacent regulated genes (i.e., TF SAR genes).

### 3.2. TFs Tend to Be in the Same Cellular Component with Binding TFs of their SAR Genes

**Figure 2:**
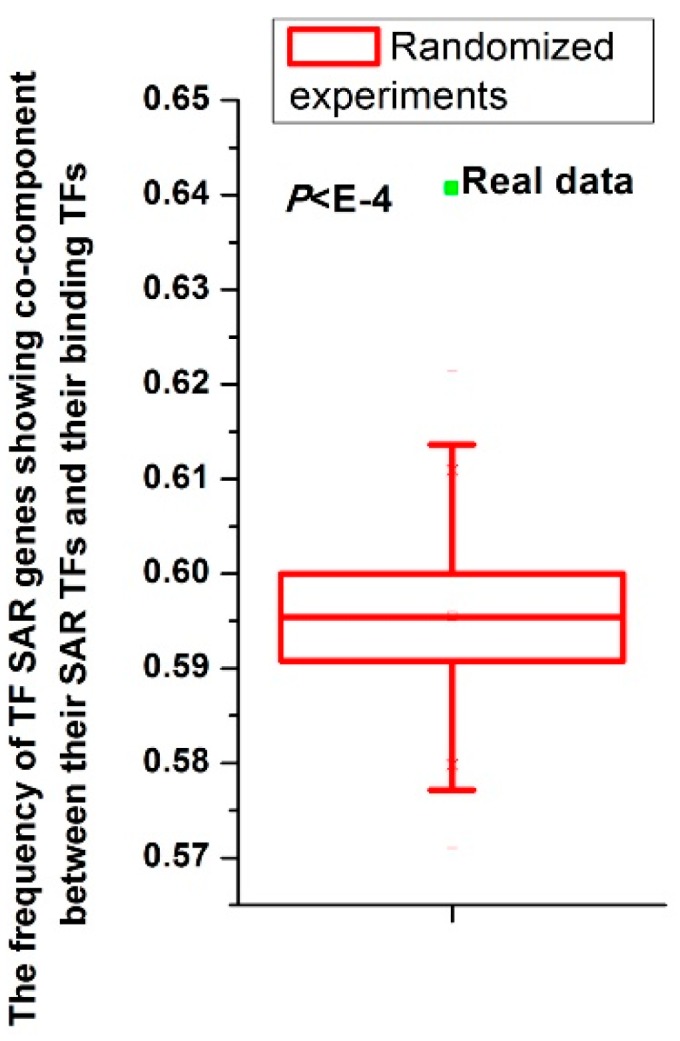
For all TFs, TFs tend to be in the same cellular component with binding TFs of their SAR genes. Distributions of the frequencies of pairs between TFs and their SAR genes showing cellular co-component between their SAR TFs and binding TFs of TF SAR genes. The dot was for the realistic data (3339/5210 = 64.09%), while the box plot depicted the distributions for 10,000 randomized experiments. The statistical significance was indicated.

I next sought to understand how TFs control expression of their SAR genes without direct DNA binding. TFs generally regulate gene expression in protein complexes. I asked whether TFs regulate their SAR genes by being in the same complexes with TFs directly binding these genes. Using budding yeast GO data [41] and analyzing all pairs between TFs and their SAR genes, I found that most (~64%) of the pairs show the following property: the TF and binding TFs of the TF SAR gene belong to the same cellular component. I generated random pairs to test the statistical significance (see details in Materials and Methods section). I repeated the randomized experiment 10,000 times to calculate the P value. The frequency of actual pairs between TFs and their SAR genes showing the above property was higher than those of all randomized experiments (*P* < 10^−4^, Figure 2). This result indicated that though TFs regulate their SAR genes without direct DNA binding, they tend to be in the same cellular components with TFs that directly bind these genes. 

### 3.3. TF SAR Genes Show Co-expression with Genes Bound by the Corresponding TF

**Figure 3:**
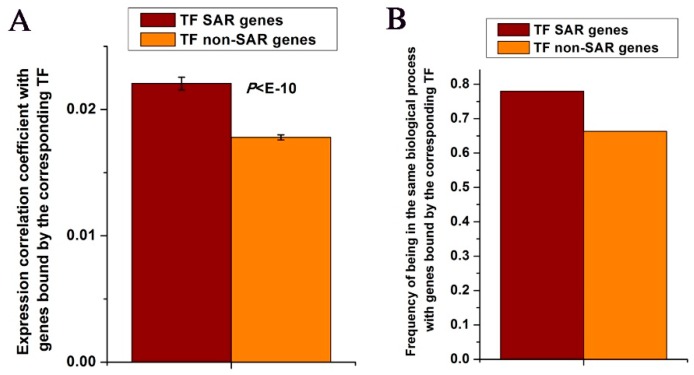
TF SAR genes tend to be involved in the same biological process with genes bound by the corresponding TF. (**A**) Median values that correspond to expression correlation coefficient between TF SAR genes (or TF non-SAR genes) and genes bound by the corresponding TF were shown. Error bars were calculated by bootstrapping. The statistically significant value calculated from the Mann-Whitney U-test was indicated. (**B**) Values that correspond to the frequencies of all pairs between TFs and their SAR genes (or TF non-SAR genes) that are involved in the same biological process with genes bound by the corresponding TFs were shown.

Genes bound by similar TFs generally show gene co-expression. I have above shown that TF SAR genes and genes bound by the corresponding TF tend to be linked by the protein complexes that include the corresponding TF. I asked whether these interactions could strengthen co-expression of TF SAR genes with genes bound by the corresponding TF. As a control, I also examined the co-expression of TF-KO genes that are not spatially adjacent to the corresponding TF bound genes (i.e., TF non-SAR genes). Using a combined gene expression data set on 112 time points in budding yeast [42,43,44], I found that TF SAR genes showed higher co-expression with genes bound by the corresponding TF (*P* < 10^−10^, Mann-Whitney U-test, Figure 3A). I next tested whether this co-expression pattern has functional consequence. Using GO data and analyzing all TF SAR genes [41], I found that ~78% of TF SAR genes were involved in the same biological process with genes bound by the corresponding TF, whereas only ~66% of TF non-SAR genes showed this feature (Figure 3B). These results suggest that TFs can strengthen their regulation through spatial proximity even though no direct DNA binding.

### 3.4. TF SAR Genes Lack DNA Motifs of the Corresponding TF

**Figure 4:**
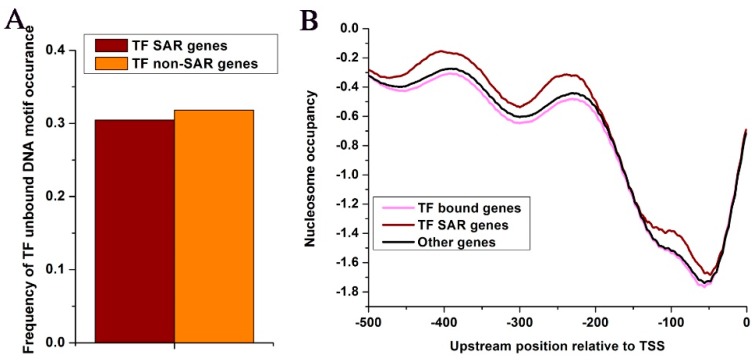
TF SAR genes lack DNA motifs of the corresponding TF. (**A**) Values that correspond to the frequencies of pairs between all TFs and their SAR genes (or TF non-SAR genes) that have DNA motifs of the corresponding TFs in promoter regions were shown. (**B**) Average nucleosome occupancy profiles in promoter regions were plotted for all TF bound genes and all TF SAR genes. To avoid confusion, we excluded genes that are both TF bound genes and TF SAR genes.

I next investigated into why TF SAR genes are not directly bound by the corresponding TF. One possible explanation is that these genes lack DNA motifs of the corresponding TF. Using the list of motifs unbound by various TFs identified in a previous study [46], I found that only ~30% of all TF SAR genes had DNA motifs for potential binding of the corresponding TF, which was comparable with the frequency (~32%) of TF non-SAR genes (Figure 4A). Another possible explanation for the unbinding of TF SAR genes by the corresponding TF is that nucleosomes compete with TFs for DNA binding. Using genome-wide nucleosome occupancy [45], I found that TF SAR genes had higher nucleosome occupancy in promoter regions ([−500, −1] relative to TSS) (*P* < 10^−10^ for TF bound genes, *P* < 10^−6^ for the other genes, Mann-Whitney U-test, Figure 4B). Moreover, TF SAR genes were bound by fewer TFs than the other genes (*P* < 10^−115^, Mann-Whitney U-test). These results together suggest that inherent DNA sequence and chromatin structure of TF SAR genes inhibit DNA binding of the corresponding TF.

### 3.5. TF-KO Genes Show Nuclear Colocalization with TF Bound Genes that Have High Corresponding TF-DNA Binding Affinities

**Figure 5:**
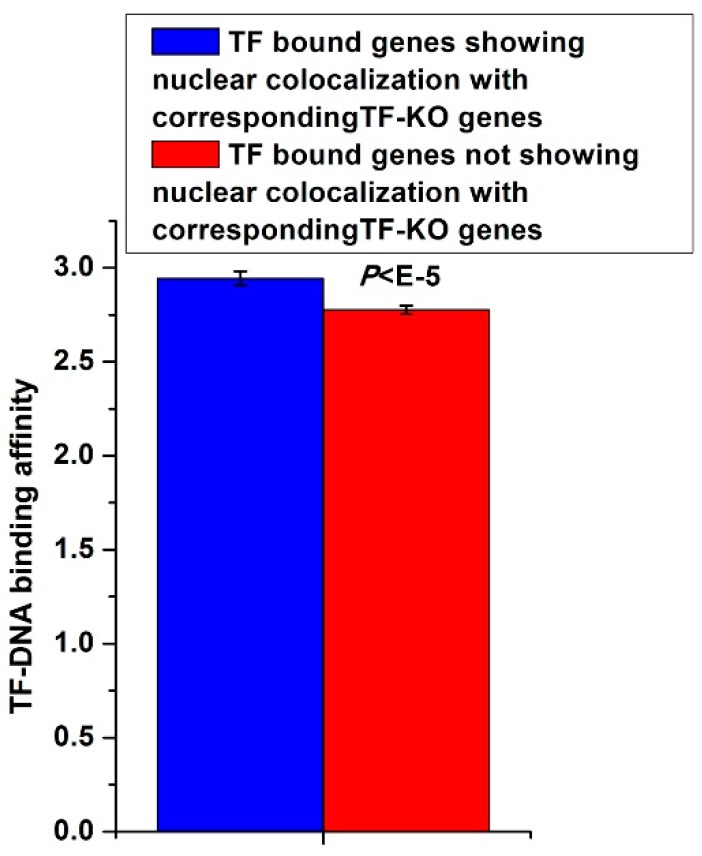
TF-KO genes show nuclear colocalization with TF bound genes that have higher corresponding TF-DNA binding affinities. For all TF bound genes that show nuclear colocalization with the corresponding TF-KO genes, the DNA binding affinities of corresponding TFs were shown. For all TF bound genes not showing nuclear colocalization with the corresponding TF-KO genes, the DNA binding affinities of corresponding TFs were shown. Error bars were calculated by bootstrapping. The statistical significant value calculated from Mann-Whitney U-test was indicated.

I asked whether TF bound genes, which show nuclear colocalization with the corresponding TF-KO genes, have some specific properties. As these TF molecules regulate their non-direct-bound genes by binding other genes that are spatially adjacent to these non-direct-bound genes, a natural question was to ask whether this regulatory mode requires stable TF-DNA binding. I found that TF-DNA binding events in this regulatory mode had higher binding affinities than other TF-DNA binding events (*P* < 10^−115^, Mann-Whitney U-test, Figure 5). For each TF, I calculated pair-wise Pearson expression correlation coefficient among its bound genes that show nuclear colocalization with its knockout affected genes. For each TF, I also calculated pair-wise Pearson expression correlation coefficient among its bound genes not showing nuclear colocalization with its knockout affected genes. These two types of TF-DNA binding events showed comparable expression correlation levels (*P* = 0.81, Mann-Whitney U-test). These results indicate that though TF bound genes that show nuclear colocalization with TF-KO genes have high DNA binding affinities of the corresponding TF, these high affinities do not result in high co-regulation of genes bound by the corresponding TF. Although the difference in Figure 5 is somewhat marginal, it still implied that the high TF-DNA binding affinities on these genes is associated with their spatially proximal TF-KO genes.

## 4. Discussion

**Figure 6:**
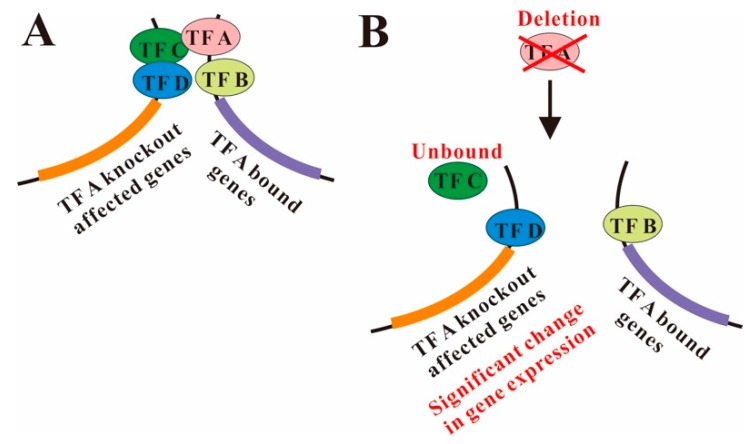
A model for TF regulation of genes through nuclear colocalization without direct DNA binding. (**A**) Although without direct DNA binding, TF A still regulates its knockout affected genes through being in the same protein complex with TFs (e.g., TF C) that directly bind the gene. (**B**) When the knockout of TF A, it may influence the TF recruitment or binding (e.g., TF C) of its knockout affected gene, consequently affecting gene expression.

Here, I investigated into the mechanism for the small overlap of TF-KO genes with genes bound by the corresponding TF in budding yeast. Previous studies have revealed that TF-KO genes or TF bound genes show spatially proximity, respectively [30,47]. To the best of my knowledge, there was no previous study revealing spatial relationship between TF-KO genes and TF bound genes. Focusing on TF-KO genes not bound by the corresponding TFs, I found that a statistically significant number of TF-KO genes show nuclear colocalization with genes bound by the corresponding TF on a genome scale. For these TF-KO genes showing nuclear proximity with their corresponding TFs, their direct binding TFs tend to be in the same cellular component with their corresponding TF. Moreover, these TF spatially adjacent knockout affected genes show stronger co-regulation (co-expression and biological co-process) with genes bound by the corresponding TF, compared with the co-regulation between TF spatially non-adjacent knockout affected genes and genes bound by the corresponding TF. Based on these results, it is most likely that though the corresponding TFs do not directly bind TF-KO genes, the TFs are in the protein complexes in which some other TFs directly bind the TF-KO genes (Figure 6A). After the knockout of the corresponding TFs, the components in the protein complexes that directly bind TF-KO genes are altered, TF-KO genes are thus differentially expressed (Figure 6B).

My results indicate that TFs regulate their knockout affected genes through inter- and intra-chromosomal interactions without direct DNA binding. I have revealed that two possible explanations for the use of this regulatory mode are the lack of corresponding TF bound motifs, and high nucleosome occupancy that inhibits TF binding in promoter regions. I asked whether this regulatory mode resulted in high gene expression variability. However, TF spatially adjacent knockout affected genes show comparable gene expression variability with the other genes (*P* = 0.42, Mann-Whitney U-test, data not shown).

I asked whether TF direct bound genes that are spatially proximal to genes affected by knockout of the corresponding TF show different properties compared with other TF bound genes. The former type of TF-DNA binding may regulate both TF direct bound genes and TF-KO genes. Our results indicate that this dual regulatory mode does not influence co-regulation of TF direct bound genes: co-expression between TF direct bound genes that are spatially proximal to genes affected by knockout of the corresponding TFs are similar to those between the other genes bound by the corresponding TFs. In addition, this dual regulatory mode requires high TF-DNA binding affinity. 

An interesting result in this study is that the number of TF direct bound genes showing nuclear colocalization with genes affected by knockout of the corresponding TFs is not statistically significant. In this analysis, I considered only whether TF direct bound genes show nuclear colocalization with corresponding TF-KO genes, but not the exact number of corresponding TF-KO genes that TF direct bound genes show nuclear colocalization with. This result seems inconsistent with another my observation that the number of TF-KO genes showing nuclear colocalization with genes bound by the corresponding TFs is statistically significant. A possible explanation to reconcile the inconsistency is that the number of spatially adjacent corresponding TF-KO genes per TF bound gene is higher than that of spatially adjacent corresponding TF bound genes per TF knockout affected gene (Figure S3). Note that the total number of inter- and intra-chromosomal interactions between TF bound genes and TF-KO genes is the same for the two cases above. As a result, the number of TF direct bound genes showing nuclear colocalization with genes affected by knockout of the corresponding TFs is lower than that of TF-KO genes showing nuclear colocalization with genes bound by the corresponding TFs.

## Supplementary Materials

The following are available online at https://www.mdpi.com/2073-4409/8/7/754/s1, Figure S1: Same as Figure 1, but for another set of TF-KO genes identified by Hu et al., Figure S2: For all TFs, TF bound genes do not show nuclear colocalization with corresponding TF-KO genes., Figure S3: The distribution of the numbers of spatially adjacent corresponding TF-KO genes per TF bound gene was shown, Table S1: The frequencies of TF-KO genes showing inter- and intra-chromosomal colocalization with corresponding TF bound genes were shown for individual TFs.

## Abbreviations

TF-KO genes	TF knockout affected genes
inter-chr colocalization	inter-chromosomal colocalization
intra-chr colocalization	intra-chromosomal colocalization
TF SAR genes	TF spatially adjacent regulated genes
SAR TFs	spatially adjacent regulatory TFs
