# Supplementary material for: Transcription Factors Indirectly Regulate Genes through Nuclear Colocalization
Source: Cells. 2019 Jul 20;8(7):754. doi: 10.3390/cells8070754 (PMC6678861; doi:10.3390/cells8070754)
Supplement: Supplementary file 1 [file cells-08-00754-s001.pdf]

Supplementary Material

Transcription factors indirectly regulate genes through nuclear colocalization

Zhiming Dai[1,2§]

[1]School of Data and Computer Science, Sun Yat-Sen University, Guangzhou 510006, China
[2]Guangdong Province Key Laboratory of Big Data Analysis and Processing, Sun Yat-Sen University, Guangzhou 510006, China

[§]daizhim@mail.sysu.edu.cn

Figures. S1 to S3
Table S1

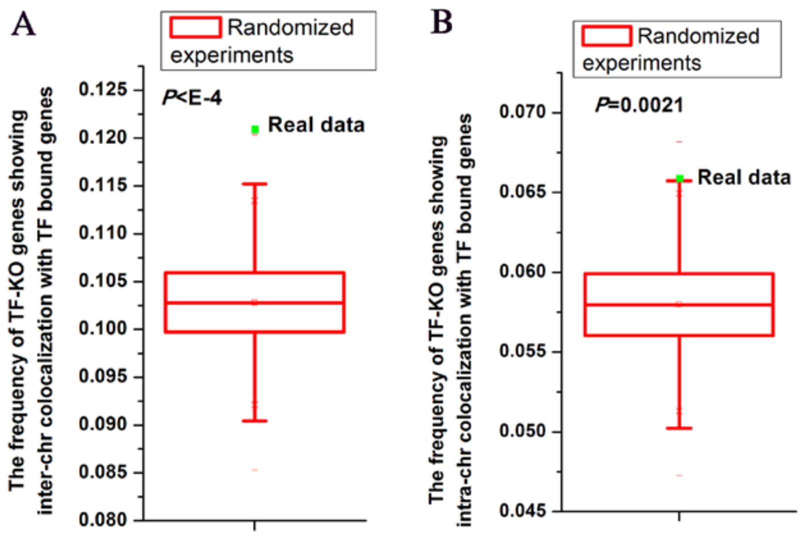

Figure S1. Same as Figure 1, but for another set of TF-KO genes identified by Hu *et al.*. The dots were for the realistic data (797/6,283=12.69%, 415/6,283=6.60%), while the box plots depicted the distributions for 10,000 randomized experiments. The statistical significance were indicated.

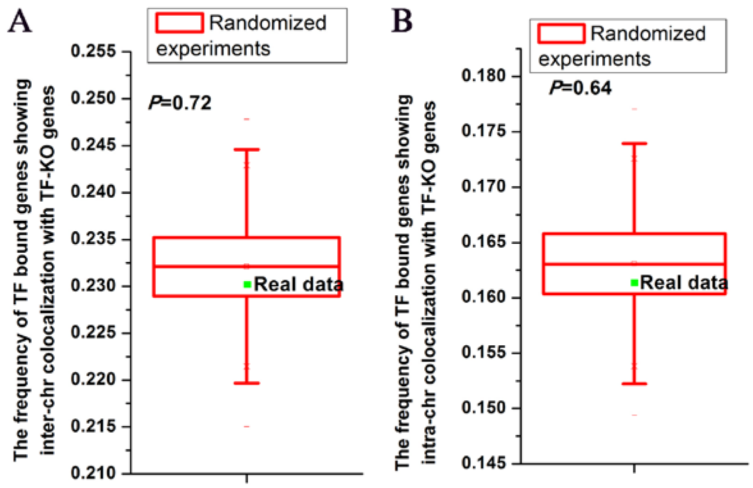

Figure S2. For all TFs, TF bound genes do not show nuclear colocalization with corresponding TF-KO genes. Distributions of the frequencies of pairs between TF and its bound genes that show inter-chromosomal (i.e. inter-chr) colocalization (A) and intra-chromosomal (i.e. intra-chr) colocalization (B) with genes whose expression were affected by knockout of the corresponding TF. The dots were for the realistic data(1,970/8,555=23.03%, 1,388/8,555=16.22%), while the box plots depicted the distributions for 10,000 randomized experiments. The statistical significance were indicated.

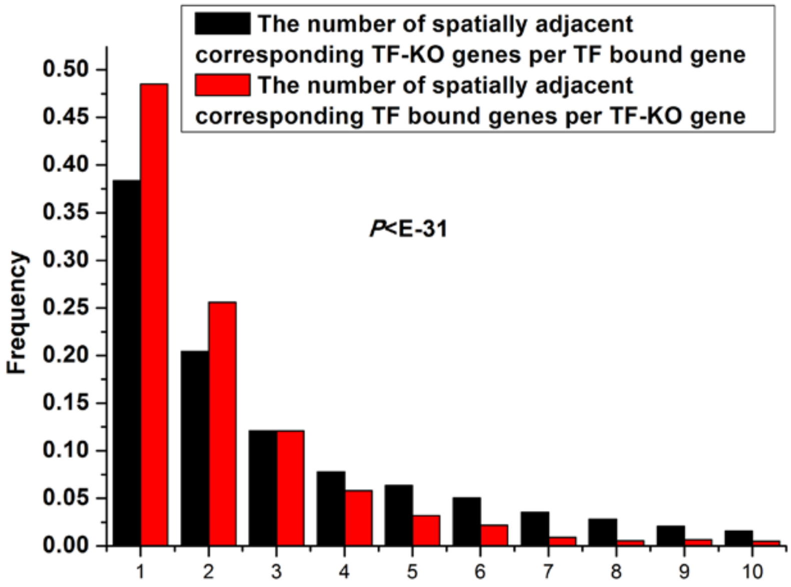

Figure S3. The distribution of the numbers of spatially adjacent corresponding TF knockout affected genes per TF bound gene was shown. The distribution of the numbers of spatially adjacent corresponding TF bound genes per TF knockout affected gene was also shown. The statistical significant value calculated from Mann-Whitney U-test was indicated.

Table S1 The frequencies of TF-KO genes showing inter- and intra-chromosomal colocalization with corresponding TF bound genes were shown for individual TFs.

TF	The number of TF-KO genes	The number of TF-KO genes showing inter-chromosomal colocalization with TF bound genes	The frequency of TF-KO genes showing inter-chromosomal colocalization with TF bound genes	The number of TF-KO genes showing intra-chromosomal colocalization with TF bound genes	The frequency of TF-KO genes showing intra-chromosomal colocalization with TF bound genes
ABF1	693	196	0.28282828	120	0.17316
ACE2	519	64	0.12331407	37	0.071291
ADR1	91	11	0.12087912	2	0.021978
AFT2	246	13	0.05284553	14	0.056911
ARG80	293	24	0.08191126	13	0.044369
ARG81	261	11	0.04214559	7	0.02682
ARO80	331	39	0.11782477	14	0.042296
ARR1	184	13	0.07065217	9	0.048913
ASK10	115	9	0.07826087	6	0.052174
AZF1	62	8	0.12903226	2	0.032258
CAD1	303	26	0.08580858	15	0.049505
CBF1	529	69	0.13043478	35	0.066163
CHA4	117	7	0.05982906	4	0.034188
CIN5	599	130	0.21702838	75	0.125209
CUP9	983	60	0.06103764	27	0.027467
DAL80	106	4	0.03773585	3	0.028302
DAL81	123	7	0.05691057	9	0.073171
DAL82	652	66	0.10122699	15	0.023006
DAT1	213	15	0.07042254	6	0.028169
DIG1	267	38	0.1423221	11	0.041199
EDS1	132	11	0.08333333	5	0.037879
FKH1	82	14	0.17073171	14	0.170732
FKH2	386	60	0.15544041	45	0.11658
FZF1	108	14	0.12962963	8	0.074074
GAL3	84	11	0.13095238	2	0.02381

GAL4	1067	113	0.1059044	39	0.036551
GAT1	58	3	0.05172414	0	0
GAT3	166	23	0.13855422	6	0.036145
GCN4	264	41	0.15530303	26	0.098485
GCR2	811	83	0.10234279	33	0.040691
GLN3	584	39	0.06678082	22	0.037671
GTS1	409	29	0.07090465	13	0.031785
GZF3	89	8	0.08988764	5	0.05618
HAC1	43	5	0.11627907	3	0.069767
HAL9	161	16	0.09937888	7	0.043478
HAP3	120	4	0.03333333	10	0.083333
HAP4	156	10	0.06410256	5	0.032051
HAP5	259	15	0.05791506	10	0.03861
HIR1	237	16	0.06751055	13	0.054852
HIR2	226	8	0.03539823	6	0.026549
HIR3	363	42	0.11570248	18	0.049587
HMS2	67	13	0.19402985	5	0.074627
HOG1	209	5	0.02392344	5	0.023923
HSF1	1059	63	0.05949008	19	0.017941
INO2	139	13	0.09352518	8	0.057554
INO4	190	36	0.18947368	23	0.121053
IXR1	487	34	0.0698152	13	0.026694
KSS1	167	10	0.05988024	9	0.053892
LEU3	121	8	0.0661157	4	0.033058
MAC1	254	38	0.1496063	15	0.059055
MBP1	92	15	0.16304348	7	0.076087
MCM1	1284	112	0.08722741	78	0.060748
MDS3	37	3	0.08108108	0	0
MET18	222	11	0.04954955	8	0.036036
MET28	87	8	0.09195402	7	0.08046
MET31	54	7	0.12962963	1	0.018519
MET32	33	4	0.12121212	5	0.151515
MGA1	195	36	0.18461538	11	0.05641
MIG1	199	17	0.08542714	10	0.050251
MIG2	105	8	0.07619048	2	0.019048
MIG3	164	6	0.03658537	2	0.012195
MOT3	318	31	0.09748428	9	0.028302
MSN4	101	10	0.0990099	3	0.029703
MTH1	337	34	0.10089021	14	0.041543
NDT8	517	73	0.14119923	20	0.038685

0					
NRG1	124	11	0.08870968	9	0.072581
OAF1	301	33	0.10963455	29	0.096346
PDR1	285	33	0.11578947	22	0.077193
PDR3	210	11	0.05238095	7	0.033333
PHD1	263	53	0.20152091	18	0.068441
PHO2	605	78	0.12892562	45	0.07438
PHO4	551	99	0.17967332	53	0.096189
PIP2	1052	122	0.11596958	46	0.043726
PPR1	164	27	0.16463415	13	0.079268
RAP1	1040	145	0.13942308	86	0.082692
RCO1	456	45	0.09868421	20	0.04386
RCS1	707	45	0.06364922	7	0.009901
REB1	500	113	0.226	70	0.14
RFX1	186	14	0.07526882	5	0.026882
RGM1	251	3	0.01195219	0	0
RIM101	483	26	0.05383023	12	0.024845
RLM1	54	1	0.01851852	0	0
RLR1	61	4	0.06557377	2	0.032787
RME1	113	14	0.12389381	6	0.053097
ROX1	165	30	0.18181818	17	0.10303
RPN4	356	62	0.1741573	30	0.08427
RTG1	141	7	0.04964539	7	0.049645
RTG3	459	22	0.04793028	4	0.008715
SIG1	91	9	0.0989011	4	0.043956
SIP4	140	10	0.07142857	6	0.042857
SKN7	252	24	0.0952381	20	0.079365
SKO1	304	8	0.02631579	6	0.019737
SMK1	112	1	0.00892857	2	0.017857
SMP1	57	13	0.22807018	7	0.122807
SNT2	104	13	0.125	2	0.019231
SPT2	335	14	0.04179104	12	0.035821
SPT23	92	9	0.09782609	1	0.01087
STB1	314	37	0.11783439	15	0.047771
STB2	173	20	0.11560694	9	0.052023
STB4	50	3	0.06	5	0.1
STB5	399	39	0.09774436	17	0.042607
STP1	491	25	0.0509165	9	0.01833
STP2	160	20	0.125	4	0.025
STP4	162	7	0.04320988	1	0.006173
SUM1	462	16	0.03463203	13	0.028139
SUT1	168	31	0.18452381	21	0.125

SWI4	402	54	0.13432836	36	0.089552
SWI5	275	45	0.16363636	24	0.087273
SWI6	427	71	0.16627635	24	0.056206
TEC1	616	29	0.04707792	11	0.017857
TYE7	1093	87	0.07959744	45	0.041171
UME6	599	68	0.11352254	32	0.053422
UPC2	73	7	0.09589041	3	0.041096
USV1	82	4	0.04878049	0	0
WAR1	56	10	0.17857143	2	0.035714
WTM1	181	10	0.05524862	1	0.005525
YAP1	99	15	0.15151515	3	0.030303
YAP3	293	26	0.0887372	10	0.03413
YAP5	133	14	0.10526316	11	0.082707
YAP6	64	8	0.125	3	0.046875
YDR026c	86	13	0.15116279	2	0.023256
YDR049W	180	28	0.15555556	15	0.083333
YDR266c	200	25	0.125	9	0.045
YER051w	102	9	0.08823529	0	0
YER130C	155	22	0.14193548	12	0.077419
YFL044C	240	14	0.05833333	15	0.0625
YFL052w	84	8	0.0952381	4	0.047619
YHP1	180	18	0.1	8	0.044444
YJL206C	120	8	0.06666667	4	0.033333
YKR064W	115	7	0.06086957	7	0.06087
YLR278C	417	43	0.10311751	20	0.047962
YML081W	211	9	0.04265403	8	0.037915
YOX1	151	21	0.13907285	9	0.059603
YRR1	142	21	0.14788732	11	0.077465
ZAP1	122	4	0.03278689	3	0.02459
ZMS1	138	2	0.01449275	1	0.007246
